# Massive hemothorax from injury of an anonymous vein after intercostal chest drain placement: A case report

**DOI:** 10.1016/j.amsu.2021.102854

**Published:** 2021-09-13

**Authors:** Motohiro Kikukawa, Akira Kuriyama

**Affiliations:** Emergency and Critical Care Center, Kurashiki Central Hospital, Japan

**Keywords:** Hemothorax, Intercostal chest drain, Complication, ICD, intercostal chest drain: CT, computed tomography: ICU, intensive care unit

## Abstract

**Background:**

Placement of an intercostal chest drain (ICD) is an essential procedure in the management of patients with chest injuries. However, ICD placement can have complications. Here, we report a case of massive hemothorax due to injury of an anonymous vein associated with ICD placement.

**Case presentation:**

An 84-year-old man with chronic right pleural effusion from pleuroperitoneal communication presented with dyspnea after a fall. An ICD was placed in the right seventh intercostal area on the middle axillary line. He later complained of chest pain and dyspnea again due to right pneumothorax, and massive hemorrhagic pleural effusion was drained from an additionally placed ICD. A contrast-enhanced computed tomography scan showed that bleeding from the parietal pleura traveled along the first ICD and dropped into the intrapleural space. Intraoperatively, there was intramuscular venule bleeding from the right serratus anterior muscle, which was then ligated to stop the bleeding.

**Discussion:**

An optimal area to place an ICD is termed the “safety triangle”, which is determined by the pectoralis major, latissimus dorsi, and the level of the nipples and the base of the axilla. In this case, the ICD was placed in the seventh intercostal area, which is more than two intercostal distances inferior to the ‘safety triangle’

**Conclusions:**

This case suggested that, even though the vessel was small, a massive, life-threatening hemothorax can occur if an injury is caused by ICD placement. Knowledge of the anatomy necessary for placing an ICD should be reinforced.

## Highlights

• Intercostal chest drain (ICD) placement is not without complications; major ones include visceral or vascular injuries.
• Intercostal and thoracoacromial arteries could be a source of bleeding associated with ICD placement.
• An anonymous vein in the serratus anterior muscle can be injured during ICD insertion which could lead to massive hemothorax.
• The "safety triangle" should be reminded to avoid complications due to ICD placement.

## 1 Introduction

Intercostal chest drain (ICD) placement is an essential procedure for management of chest injuries. However, complications occur in approximately 20% of ICD placements [1]. The severity of complications associated with ICD placement varies. Minor complications include kinking, subcutaneous insertion, shallow placement, and slipping off of the intrapleural space, while major complications include visceral or vascular injuries [2]. We encountered a case of massive hemothorax due to the injury of an anonymous vein associated with ICD placement. This case was reported according to SCARE statement [3].

## 2 Case presentation

An 84-year-old man with nephrotic syndrome and congestive heart failure was transferred to our hospital after falling from his bed and hitting his chest. He had chronic ascites and right pleural effusion from pleuroperitoneal communication. He had daily taken azosemide 120 mg, spironolactone 25 mg, mefruside 75 mg, and tolvaptan 15 mg.

On arrival, his vital signs were as follows: blood pressure, 178/64 mmHg; pulse rate, 70 beats/min; respiration rate, 21 breaths/min; oxygen saturation, 92% on room air; and body temperature, 36.6 °C. He complained of right chest pain and dyspnea. Auscultation revealed normal heart sounds and diminished breath sounds on the right side. There was swelling around the right acromioclavicular joint. A computed tomography (CT) scan revealed right clavicle fracture and massive pleural effusion in the right intrapleural space. The cause of his dyspnea was considered the pleural effusion. An ICD was placed by an emergency medicine fellow (postgraduate year 4) in the right seventh intercostal area on the middle axillary line, and approximately 1000 mL of serous pleural effusion was drained. The dyspnea improved, and the ICD was temporarily clamped.

**Fig. 1:**
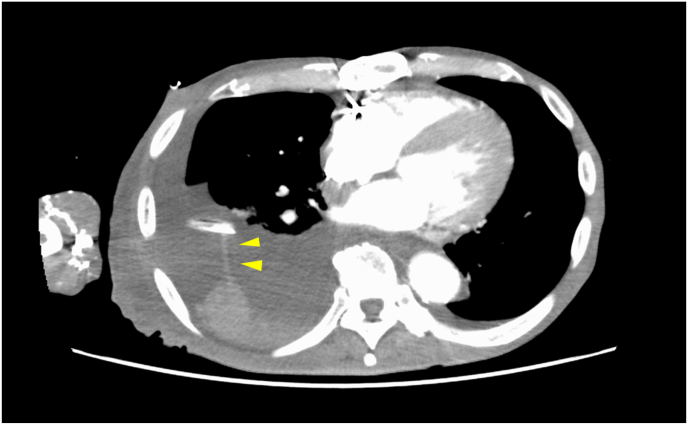
A contrast-enhanced CT scan showed that bleeding from the parietal pleura traveled along the first ICD and dropped into the intrapleural space.

He complained of chest pain and dyspnea again several hours later. Chest radiography suggested right pneumothorax. We placed another ICD in the right third intercostal space on the midclavicular line. Approximately 1500 ml of hemorrhagic pleural effusion was drained from the second ICD immediately after the placement. We clamped the second ICD and initiated transfusion. A contrast-enhanced CT scan showed the bleeding from the parietal pleura traveled along the first ICD and dropped into the intrapleural space (Fig. 1). We intubated the patient and two surgeons (postgraduate year 10 and 14, respectively) performed a right lateral thoracotomy in the intensive care unit (ICU).

There was intramuscular venule bleeding from the right serratus anterior muscle which was ligated to stop the bleeding. We separated the intercostal muscles and performed a right anterolateral thoracotomy to look for another bleeding source. After clearing the intrapleural space of hemorrhagic effusion and clots, no bleeding source was identified. We concluded that the bleeding from the serratus anterior muscle caused the massive right hemothorax.

We removed the first ICD at the end of the surgery. The patient's respiratory and hemodynamic states improved, and the patient was extubated and discharged from the ICU on the fourth day after admission. On the seventh day, the second ICD was removed. The patient was discharged on the 73rd day after admission.

## 3 Discussion

Placement of ICD is a common procedure for thoracic injury treatment and has been standardized as applied in the Advanced Trauma Life Support® (ATLS®) training course. The procedure of ICD placement appears to be relatively safe, but it can lead to serious complications. Frequent complications from ICD placement, such as kinking or bending, are usually minor [2]. However, rare complications, including visceral (2%) and vascular injury (2%), can be fatal [2].

An optimal area to place an ICD is termed the “safety triangle”, which is surrounded by the lateral edges of the pectoralis major and latissimus dorsi and is superior to the level of the nipples and inferior to the base of the axilla. Among cases with ICD-associated complications, not including visceral or vascular injury, 41% of ICDs were placed outside the “safety triangle” [2]. In our case, the ICD was placed in the seventh intercostal area, which is more than two intercostal distances inferior to the ‘safety triangle’.

Previous reports suggest that the intercostal and thoracoacromial arteries could be a source of bleeding associated with ICD placement. A case report suggested that bleeding from anonymous vessels in the chest wall after an invasive procedure could be fatal [4]. In contrast, few case reports have described vascular injury of the serratus anterior muscle. One report suggested that some vessels communicate between the serratus anterior muscle slips [5]. We suspect that this vessel might have been damaged in our patient. More knowledge on the anatomical distribution of these vessels is needed when placing an ICD.

We clamped the ICD to prevent re-expansion pulmonary edema. This might have masked the bleeding into the intrapleural space, leading to delayed detection of the massive bleeding in our patient. Vital signs and the amount of bleeding after de-clamping should be monitored for early detection of massive bleeding.

Previous studies have suggested that physician experience is associated with fewer complications and that the risk of complications is higher when young and inexperienced physicians perform the procedure [6,7]. In our case, an emergency medicine fellow (postgraduate year 4) placed the ICD in this patient. Given the anatomical site of ICD placement, the physician might have had limited knowledge and experience of ICD. Thus, attending physicians, if present, should review each young physician's experience with and knowledge of the procedure and supervise the procedure when necessary.

Physician knowledge of and experience with ICD vary depending on the type and intensity of the training they have received. Thus, training should be provided to reinforce knowledge and supplement the experience of young physicians regarding ICD. Studies are warranted to investigate the types of training that could reduce the incidence of complications related to ICD or the intensity and duration of training needed for safe ICD placement.

## 4 Conclusion

Massive hemothorax due to an anonymous venous injury can occur during ICD insertion, even if the injured vessel is small. Using anatomical knowledge of the “safety triangle” can help avoid complications.

## Ethical approval

Not required for case reports in Japan. We obtained written informed consent.

## Sources of funding

There are no sources of funding.

## Author contributions

MK and AK looked after the patients, wrote and revised the draft, and approved the submission the current manuscript.

## Informed consent

Written informed consent was obtained from the patient family for publication of this case report and accompanying images. A copy of the written consent is available for review by the Editor-in-Chief of this journal on request.

## Research registration

Not applicable.

## Provenance and peer review

Not commissioned; externally peer-reviewed.

## Declaration of interest

None to declare.

## Funding

None.

## Declaration of competing interest

The authors declare that they have no conflict of interest.

## References

[1] Hernandez M.C., El Khatib M., Prokop L., Zielinski M.D., Aho J.M. (2018). Complications in tube thoracostomy: systematic review and meta-analysis. The journal of trauma and acute care surgery.

[2] Kong V.Y., Oosthuizen G.V., Sartorius B., Keene C., Clarke D.L. (2014). An audit of the complications of intercostal chest drain insertion in a high volume trauma service in South Africa. Ann. R. Coll. Surg. Engl..

[3] Agha R.A., Franchi T., Sohrabi C., Mathew G., Kerwan A., Group S. (2020). The SCARE 2020 guideline: updating consensus surgical CAse REport (SCARE) guidelines. Int. J. Surg..

[4] Wu M.C., Liu K.T., Yeh I.J., Wu Y.H. (2018). Chest wall hematoma after central venous hemodialysis catheter insertion. The American journal of emergency medicine.

[5] Godat D.M., Sanger J.R., Lifchez S.D., Recinos R.F., Yan J.G., Godat M.R., Ramirez C.E., Matloub H.S. (2004). Detailed neurovascular anatomy of the serratus anterior muscle: implications for a functional muscle flap with multiple independent force vectors. Plast. Reconstr. Surg..

[6] Alrahbi R., Easton R., Bendinelli C., Enninghorst N., Sisak K., Balogh Z.J. (2012). Intercostal catheter insertion: are we really doing well?. ANZ J. Surg..

[7] Sritharen Y., Hernandez M.C., Haddad N.N., Kong V., Clarke D., Zielinski M.D., Aho J.M. (2018). External validation of a tube thoracostomy complication classification system. World J. Surg..

